# Staged surgical management of follicular thyroid carcinoma with extensive thrombus reaching up to right atrium – A case report

**DOI:** 10.1016/j.ijscr.2019.10.050

**Published:** 2019-11-01

**Authors:** Prashant Praksh Lad, Jateendar Kumar, Jagadish Sarvadnya, Abhijit S. Powar

**Affiliations:** a Department of Surgical Oncology, Om Sai Onco-Surgery Center, R/S no 457/10, Sugar Mill Corner, Main Road, Kasaba Bawada, Kolhapur, Maharashtra, 416006, India; b Sai Cardiac Center, 2021/B, 6th lane S.T Colony, Mahalaxminagar, Rajarampuri, Kolhapur, Maharashtra, 416008, India; c Department of Head and Neck Surgery, Om Sai Onco-Surgery Center, R/S number 457/10, Sugar Mill Corner, Main Road, Kasaba Bawada, Kolhapur, Maharashtra, 416006, India; d Department of Surgical Oncology, Om Sai Onco-Surgery Center, R/S number 457/10, Sugar Mill Corner, Main Road, Kasaba Bawada, Kolhapur, Maharashtra, 416006, India

**Keywords:** Follicular thyroid carcinoma, Extensive venous invasion, Internal jugular vein, Superior vena cava, Tumor thrombus in the atrium, Staged surgical approach

## Abstract

**Introduction:**

Differentiated thyroid carcinoma with an extensive intravenous tumor thrombus which is extending into internal jugular vein (IJV), superior vena cava (SVC) and right atrium (RA) is a rare clinical finding. We report a multimodal staged surgical approach for this life threatening complicated case.

**Presentation of case:**

A 52 year old female, presented with diffuse thyroid swelling, FNAC revealed it as follicular thyroid neoplasm. Computed tomography (CT) scan showed tumor thrombus extending into IJV, SVC and right atrium (RA). We planned complete resection of tumor in two stage operation. Initially in first stage, cardio-thoracic surgery was done to remove SVC and RA thrombus to eliminate the immediate risk of pulmonary embolism. In the second stage, neck surgery was performed to resect thyroid tumor and to perform extensive thrombectomy in the cervical veins. This patient has been followed for one year after successful surgery without recurrence.

**Discussion:**

Venous involvement by follicular thyroid carcinoma reaching to RA is a rare life threatening condition. Though there is no standard guidelines available, treatment strategies should be discussed and planned among multidisciplinary team. Intraluminal extension is not a contraindication for aggressive surgical management. It will avoid fatal pulmonary embolism, as well as improve overall survival of the patient.

**Conclusion:**

Invasion of the great vessels by thyroid carcinoma is usually associated with early relapse and poor prognosis, but if tumor in the blood vessel is resected completely, a better prognosis is possible. Two staged surgical approach is safe and gives a predictable outcome.

## Highlights

• Follicular thyroid carcinoma with venous tumor thrombus extending to right atrium.
• Two staged surgery avoids operative complexity, with better post operative outcome.
• Initially cardio-thoracic part done to eliminate the risk of pulmonary embolism.
• In second stage thyroid tumor resection with tumor thrombus extraction was done.
• 12 months recurrence free survival was observed.

## 1 Introduction

Thyroid cancers constitute 1%–5% of all malignancies worldwide and are increasing in incidence globally [1]. Differentiated thyroid cancer consists of papillary and follicular carcinoma, which is having favorable prognosis. Although follicular thyroid cancers may show microscopic vascular invasion, macroscopic venous tumor thrombus extending from cervical veins to mediastinal great veins and reaching to the right atrium (RA) is extremely rare [[1], [2], [3], [4], [5]]. Here we report a case of follicular thyroid carcinoma with tumor thrombus formation in the internal jugular vein & extending into the Superior Vena Cava (SVC) and RA; which is treated with radical intent in two stage surgery. The patient was treated successfully by a collaborative head-neck and cardiothoracic surgeons team. This case has been reported in line with the SCARE criteria [6]

## 2 Case report

**Fig. 1:**
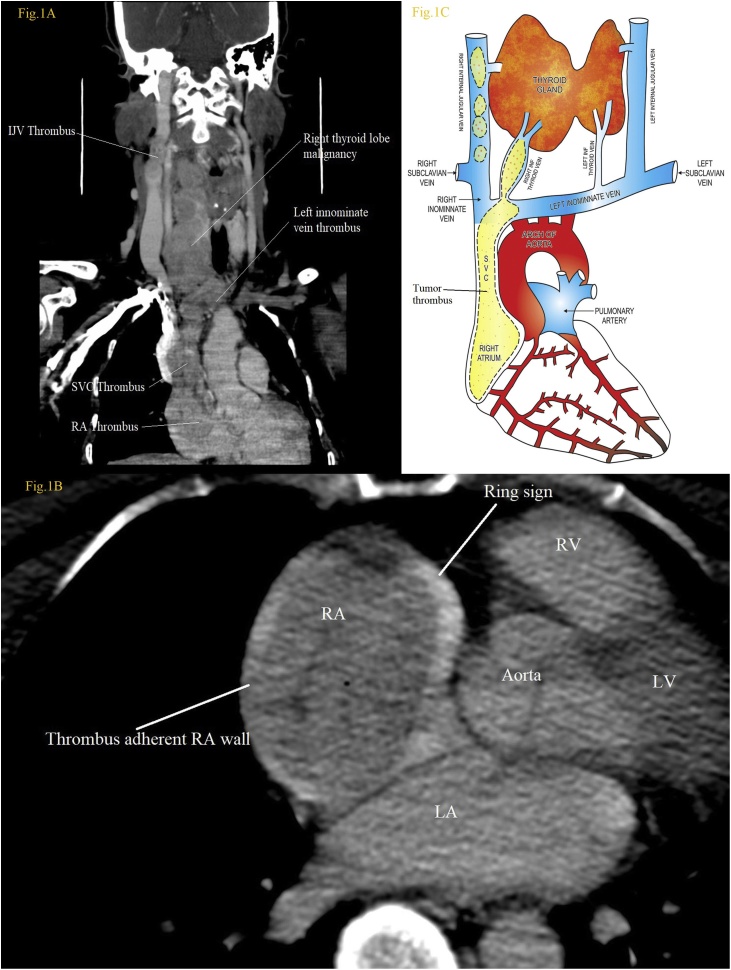
(A) Contrast enhanced CT scan coronal section showing intravenous tumor thrombus extension in the neck vein and thorax. (B) Contrast enhanced CT scan thorax axial image showing tumor thrombus in the RA with Ring sign. (C) Artistic representation of thyroid tumor (brown colored) and intravenous tumor thrombus extension in cervical veins, SVC and RA.

A 52-year-old female was referred to us with diffuse thyroid swelling since 8 months. The patient was not having dysphagia, dyspnoea or hoarseness of voice, with no significant medical illness in the past. Pertinent physical examination revealed a large lobulated mass on anterior aspect of the neck with separate fusiform swelling laterally in the right side of neck; initially it appeared to be a nodal metastasis. Her pulse was 88 beats/min, blood pressure was 136/78 mmHg and normal heart sound with no audible murmur. Fine-needle aspiration cytological examination revealed clusters of atypical follicular cells. The serum thyroglobulin level was elevated to 23,500 ng/ml. Axial post contrast computed tomography (CT) scan images of the neck demonstrated diffuse large thyroid growth involving both lobes and intra-venular extension of the malignant thrombus into the IJV, left innominate vein, SVC and reaching to the RA, occupying 80% of the total RA volume with suspicious adherence (Fig. 1B). Though CT-scan was showing extensive tumor thrombus, clinically patient was not having symptoms of SVC syndrome or cardiac failure. Taib et al. described the importance of the positive ring sign, which indicates possibility of surgical removal of thrombus by thrombectomy. Coronal reformatted post contrast enhanced CT image demonstrated a large heterogeneous enhancing soft tissue mass replacing the thyroid gland with venular extension through the superior thyroid vein into the IJV and through the inferior thyroid vein into the innominate vein. The same thrombus was extending down to reach SVC and RA (Fig. 1A, C). Furthermore on metastatic work-up there was no evidence of pulmonary embolism or distant metastasis.

2D echo-cardiography for cardiac evaluation showed tumor thrombus formation as mentioned above. Surprisingly rests of the cardiac functions were normal.

### 2.1 Operative findings

Only few cases of thyroid carcinoma with an extensive tumor thrombus in the atrium have been reported in the literature. Yamagami et al. described a single staged approach in a similar case of thyroid carcinoma with RA thrombus [8]. We anticipated complex surgical procedure, since thrombus was adherent to the anterior wall of RA. We planned surgery in two sequential stages by a team of head-neck and cardiovascular surgeons: **Stage 1**, Median sternotomy for removal of RA & SVC Thrombus. **Stage 2**, Total thyroidectomy and extensive thrombectomy of neck veins.

**Fig. 2:**
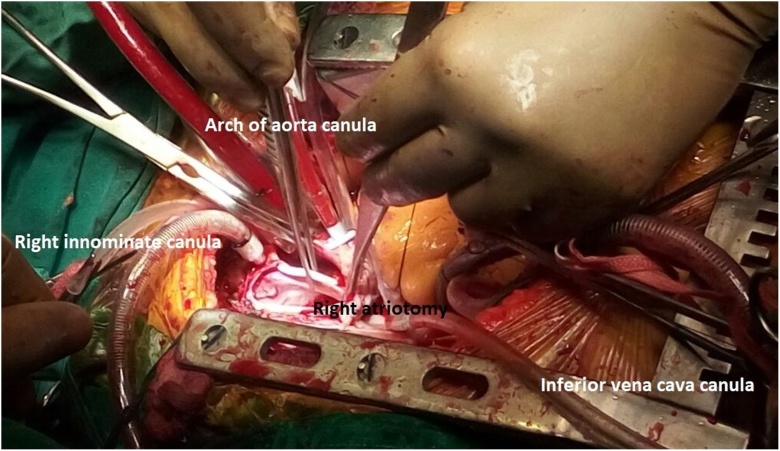
Aortic and venous cannulation for cardio-pulmonary bypass and right atriotomy being performed.

**Fig. 3:**
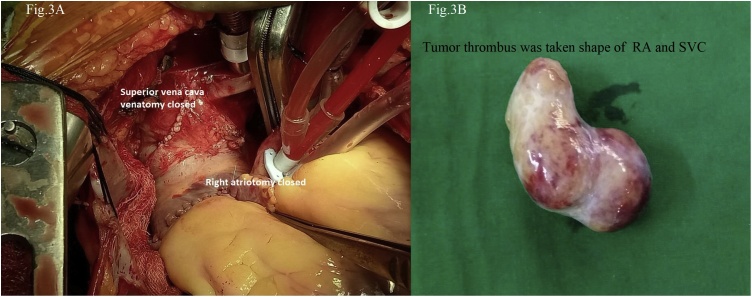
(A) Closure of SVC venotomy and right atrioromy. (B) Extracted right atrium tumor thrombus.

**Stage 1:-** To avoid the sudden death due to pulmonary embolism, it was decided to resect the intracardiac extension of the tumor before going to definitive neck surgery. Median sternotomy was performed and pericardium was opened. Patient was given heparin sodium 2 mg/kg before handling great vessels. Aortic cannulation was performed for cardio-pulmonary bypass purpose. Inferior vena cava (IVC) and right innominate vein was also cannulated with minimal manipulation of RA. Cardiopulmonary bypass was established under normothermia with IVC drain and ascending aortic perfusion. Patient’s body temperature was reduced to 22^º^C and heart beating was arrested with cardioplegia. Cardiopulmonary bypass was stopped and Total circulatory arrest achieved. Right atriotomy was performed (Fig. 2) and tumor adhesions were separated from RA wall. SVC venotomy was done by separate incision and tumor thrombus along with its extensions was delivered out through right atriotomy (Fig. 3B). Right IJV & right inferior thyroid vein openings into innominate vein were closed using intraluminal purse string sutures. SVC and RA incisions were closed separately using running sutures (Fig. 3A). Patient was slowly re-warmed to normal body temperature i.e. 37 °C and cardiopulmonary bypass was started. Total circulatory arrest time was 22 min, patient recovered without any complications.

**Fig. 4:**
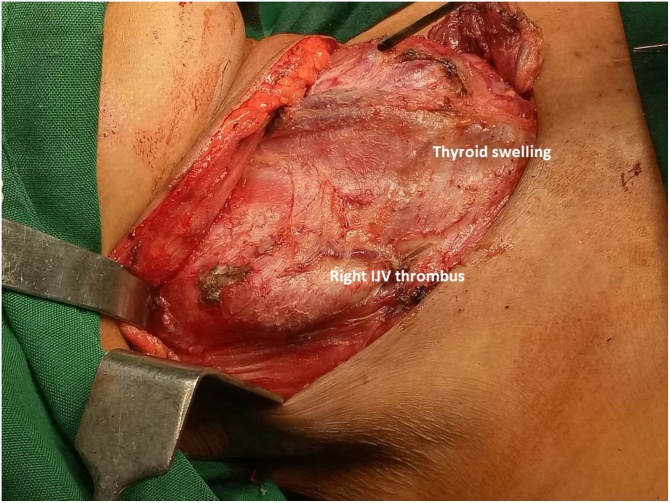
Neck surgery after exposure of anterior compartment, revealing thyroid tumor and right IJV tumor thrombus.

**Fig. 5:**
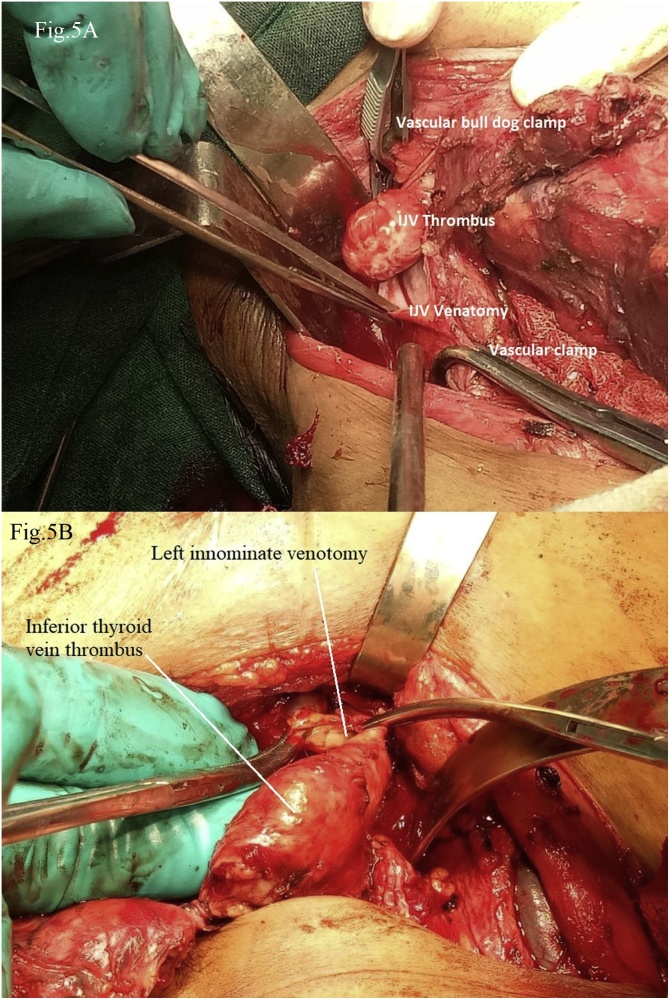
(A) IJV venotomy and tumor thrombus extraction. (B) Inferior thyroid vein tumor thrombus extending to the Left innominate vein, venotomy done for thrombus extraction.

**Stage 2:** Neck surgery for thyroid cancer was planned after complete recovery from cardio-thoracic procedure. U-shaped neck incision was given and skin flap was raised. There was diffuse thyroid swelling with distinctly seen separate lateral neck swelling on right side, which was thrombus in the IJV (Fig. 4). Prophylactic Bilateral neck and central compartment dissection was done; which was followed by dissection for total thyroidectomy. Right IJV was opened with proximal and distal control and tumor thrombus extraction was done (Fig. 5A). While dissecting Inferior Thyroid vein we found 2^nd^ tumor thrombus, so again inferior thyroid venotomy performed and tumor thrombus was extracted through the venotomy (Fig. 5B). Neck veins ostia (IJV and Inferior thyroid vein) draining into right innominate were obliterated by using purse string suture at the time of previous cardiothoracic part, so there was no fear of accidental migration of tumor thrombus into the SVC.

Total thyroidectomy specimen was removed in continuity with IJV and inferior thyroid vein thrombus. Final pathological diagnosis was follicular thyroid carcinoma with extensive intravenous tumor thrombosis. (T4b, N0, M0, stage IVA).

### 2.2 Postoperative course & follow-up

Cardiothoracic surgery part was done first followed by 4 days recovery in intensive care unit. Neck surgery was performed after 10 days of first surgery. Patient recovered without any complication and discharged from the hospital 7 days after the second surgery, total hospital stay was around 17 days.

Patient followed up closely, and after 1 month, radio-Iodine isotope-scan followed by Radioactive-iodine-131 (RAI-131) ablation with 100 mCi dose was done. Patient was monitored with serum thyroglobulin levels every third month. Patient is asymptomatic with no evidence of recurrence on RAI scan at the end of 12 months follow-up.

## 3 Discussion

Differentiated carcinoma of thyroid with haematogenus spread extending into the IJV is well reported entity [9], but spread to SVC and RA is very rare. Tumor thrombus in major vessels is reported in other malignant diseases like renal cell carcinoma, uterine carcino-sarcoma, Wilm’s tumor, testicular tumor, adrenal cortical carcinoma, lymphoma, pancreatic cancer, osteosarcoma and Ewings sarcoma [10]. Incidence of tumor thrombus of thyroid cancer is very rare and has been reported to be 0.2–3.8% [[11], [12], [13]]. In the past, Surgery for a thyroid carcinoma with extensive tumor thrombus in the internal jugular vein was first reported by Thompson et al. [2] in 1978. To the best of our Knowledge only a few case reports that describe management of thyroid carcinoma with an extensive tumor thrombus in IJV, SVC and RA have been reported in literature [8]. Proposed mechanism is, tumor invades into IJV and the high velocity flow in the cervical vein prevents the invasion of endothelium of the great vessels. This will not allow the tumor to lateralize and invade into the vessel wall [7]. Intravascular tumor extension usually propagates with intraluminal invasion by malignant cells and deposition of fibrin, leading to continued growth into the innominate vein, SVC and RA [14].

Invasion of the great vessels by thyroid carcinoma is usually associated with early relapse and poor prognosis [15], but if tumor in blood vessels is resected without residual tumor, a better prognosis is possible [1,2,11,13]. Literature also mentions survival up to 3 years after aggressive surgical resection in similar cases [16].

Similar cases receiving only palliative treatment due to difficult surgical approach resulted in poor outcome [3,17]. There are no standard guidelines available in the literature to manage this condition [18], though there are anecdotal case reports of successful surgical treatment of this condition. Yamagami et al. [8] reported a case of successful surgical resection of thyroid cancer with extensive tumor thrombus in the RA. Intraluminal extension is not a contraindication for aggressive surgical treatment in differentiated thyroid cancers due to the relatively good prognosis. Other important reasons to strongly consider surgical treatment are to avoid airway occlusion, fatal pulmonary embolism and RA obstruction [19].

Treatment strategies should be discussed among multi-disciplinary team [20]. In the literature two ways of thrombus extraction were discussed: First, by resection of mediastinal great vessels along with tumor thrombus (with or without reconstruction). Second, to do venotomy for removal of tumor thrombus with subsequent closure of venotomy. Nakano et al. [1] reported that inhibition of thyroid stimulating hormone and RAI as an adjuvant treatment is effective to improve survival.

## 4 Conclusion

Tumor thrombus usually occurs in the advanced stage of the differentiated thyroid cancer and implies that such tumors behavior is aggressive with poor prognosis. Preoperative evaluation by appropriate imaging and collaborative multi-modality approach for treatment plan is recommended. In absence of distant metastasis, median sternotomy for tumor thrombus removal and radical surgery in the neck for thyroid cancer would be the treatment of choice. Surgical treatment is definitely useful to avoid sudden fatal tumor embolism and also to improve survival. In our case, staged surgical approach was found to be safe and effective. It avoided operative complexity and perioperative morbidity with better post operative outcome.

## Sources of funding

No funding and sponsor associated with this case report.

## Ethical approval

No ethical approval required to publish this case.

## Consent

Informed consent from the patient have been taken before sending for publication.

## Author contribution

1. Dr. Prashant Lad- Cntributed to operation, follow up and writing manuscript

2. Dr. Jateendar Kumar- Cntributed to operation, follow up and writing manuscript

3. Dr. Jagadish Sarvadnya - Cntributed to operation, follow up and writing manuscript

4. Dr. Abhijith Powar - Cntributed to operation, follow up and writing manuscript

## Registration of research studies

None.

## Guarantor

Dr. Prashant P. Lad.

## Provenance and peer review

Not commissioned, externally peer-reviewed.

## Declaration of Competing Interest

I have no conflicts of interest to disclose concerning this case report.
